# Development of a Novel Nanoparticle Targeting Endothelial Cells from Patients with Pulmonary Arterial Hypertension

**DOI:** 10.3390/molecules31193511

**Published:** 2026-10-02

**Authors:** Gabriel Floarea, Nasser Assi, Maytham Ismail, Anderson Gattis, Tadanori Mammoto, Akiko Mammoto, Mathumai Kanapathipillai

**Affiliations:** 1 Department of Mechanical Engineering, University of Michigan-Dearborn, 4901 Evergreen Road, Dearborn, MI 48128, USA; 2 Department of Pediatrics, Medical College of Wisconsin, Milwaukee, WI 53226, USA; 3 Department of Pharmacology and Toxicology, Medical College of Wisconsin, Milwaukee, WI 53226, USA; 4 Department of Cell Biology, Neurobiology and Anatomy, Medical College of Wisconsin, Milwaukee, WI 53226, USA

**Keywords:** nanoparticles, targeted delivery, pulmonary arterial hypertension, endothelial cells, oxidative stress

## Abstract

Pulmonary arterial hypertension (PAH) is a devastating disease driven largely by oxidative stress and inflammation. The pulmonary arterial endothelium plays a key role in PAH pathogenesis. In this study, we developed novel poly(lactic-co-glycolic acid) (PLGA)-based lutein nanoparticles coated with a Vascular Cell Adhesion Molecule-1 (VCAM-1) antibody to target pulmonary arterial endothelial cells (PAECs) associated with PAH. Lutein, a potent antioxidant and anti-inflammatory carotenoid, served as the model drug. The lutein nanoparticles were produced by nanoprecipitation, followed by surface modification with VCAM-1 antibody. Physicochemical characterization and cellular efficacy studies were performed to assess the potential of the formulation. The studies revealed the production of nanoparticles with an average size of approximately 170 nm that exhibited specificity for PAH patient-derived PAECs (PAH-PAECs) and antioxidant and anti-inflammatory properties. Moreover, the nanoparticles did not exhibit significant cellular toxicity toward healthy PAECs. This proof-of-principle study demonstrates the therapeutic potential of targeted nanoparticles for the treatment of PAH.

## 1. Introduction

Pulmonary arterial hypertension (PAH) is a life-threatening pulmonary vascular disease [1,2]. Oxidative stress and inflammation contribute to various pathologies, including PAH [3,4,5]. PAH is associated with pulmonary fibrosis and chronic obstructive pulmonary disease (COPD), in which oxidative stress, inflammation, and endothelial dysfunction play a key role [6,7,8]. Current therapeutic approaches for PAH, including vasodilators, only partially improve symptoms and survival rates [9,10]. Therefore, improved treatment strategies are critically needed. Targeting oxidative stress and inflammation in combination with conventional therapies such as vasodilators (e.g., sildenafil) [11] could be a potential therapeutic approach for the management of PAH. Further, successfully treating PAH requires therapeutic delivery strategies that target pulmonary artery remodeling and associated pathological changes. During PAH, pulmonary arteries undergo pathophysiological changes including overexpression of adhesion molecules such as VCAM-1 and ICAM-1 [12,13,14]. These physiological changes could be utilized for targeted delivery to PAH vasculature.

Although systemic drug delivery is still widely used in treating diseases, it often results in off-target side effects, causing unintended toxicity to normal healthy tissues. Conventional treatments often suffer from poor water solubility, rapid degradation, and premature immune system clearance, requiring high doses that cause severe off-target toxicity [15]. Nanotechnology is used to overcome challenges in systemic drug delivery [16,17]. Moreover, nanomedicine has been utilized in treating various pulmonary diseases [18]. Nanocarriers can be designed to deliver drugs in a controlled-release manner, maintaining sustained drug delivery over a long period. Additionally, they can be tuned to incorporate multiple drugs and targeting moieties to deliver the drugs site-specifically at the tissue environment of interest. Various nanocarriers have been utilized in successful drug delivery applications, such as polymeric drug carriers, inorganic nanoparticles, liposomes, dendrimers, and cell-based carriers [19].

Among nanocarriers, polymeric drug carriers, in particular PLGA nanoparticles, have been widely used in drug delivery [20,21]. Furthermore, they have been approved by the FDA and are therefore ideal for translational drug delivery applications; they have been shown to have excellent biocompatibility and biodegradability and can be modified to impart additional functional properties to the particles. Moreover, PLGA nanoparticles have been shown to be capable of incorporating drug molecules with various physicochemical properties and hence are a highly versatile drug carrier [22]. Lutein is a naturally occurring carotenoid with antioxidative and anti-inflammatory properties [23,24,25] and has been shown to modulate inflammation by inhibiting inflammatory cytokines, including IL-6 and TNF-alpha [24]. Lutein is also shown to inhibit the activation of the NF-κB pathway [25], which is known to play a key role in inflammatory diseases including PAH [26]. However, lutein is extremely hydrophobic in nature and hence poses challenges in effective drug delivery [27]. Moreover, it has poor oral availability and exhibits low stability in the presence of light, temperature, and acidic conditions [28]. Recent studies have shown that the lutein drug efficacy could be improved by nanoformulation [29,30].

In this study, we developed a nanoformulation to target PAH patient-derived PAECs and deliver the antioxidant and anti-inflammatory model drug, lutein. PLGA nanoparticles encapsulated with lutein were formulated and subsequently coated with VCAM-1 antibodies for targeted delivery. Physicochemical characterization was performed to determine size, morphology, encapsulation, and coating. Cellular assays were performed to determine the cell viability, toxicity, antioxidant, and anti-inflammatory efficacy of the nanoparticles. Our findings demonstrate that the nanoparticles preferentially target ECs derived from patients with PAH compared with healthy ECs, improve cellular uptake, and exhibit antioxidant and anti-inflammatory effects. Overall, this study provides a proof-of-concept platform for targeting PAH patient ECs that could mitigate off-target effects while improving therapeutic efficacy.

## 2. Results

**Figure 1:**
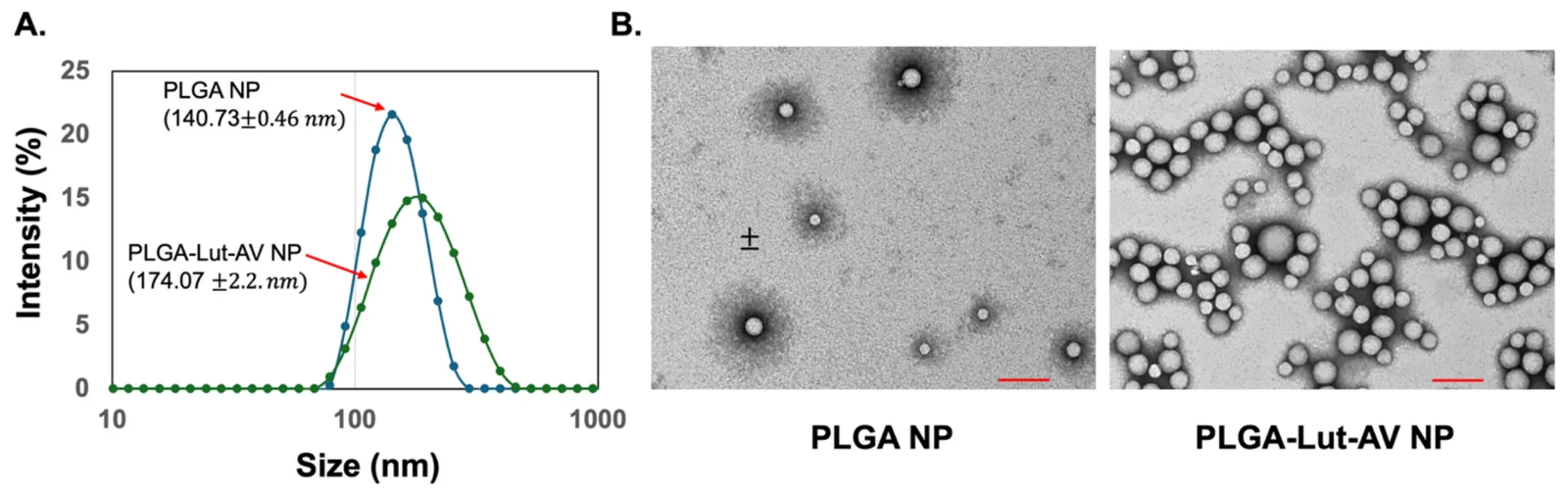
(**A**) DLS measurement of nanoparticles. Size measurements reveal particle sizes of 140 nm and 174 nm for PLGA and PLGA-Lut-AV nanoparticles, respectively. (**B**) TEM images of the nanoparticles showing the morphology and size range of PLGA and PLGA-Lut-AV nanoparticles. Scale bar: 200 nm.

First, PLGA nanoparticles alone and encapsulated with lutein were produced by nanoprecipitation. The PLGA nanoparticles encapsulated with lutein were subsequently modified with VCAM-1 to yield PLGA-Lut-AV nanoparticles. Size and morphology were characterized by DLS and TEM (Figure 1). The size measurement by DLS revealed that PLGA nanoparticles had an average size of 140.73 ± 0.46 nm and PLGA-Lut-AV nanoparticles had a size of around 174.07 ± 2.2 nm (Figure 1A). The increase in PLGA-Lut-AV nanoparticle size compared with PLGA nanoparticles alone is expected due to the incorporation of lutein and subsequent modification with the VCAM-1 antibody. The TEM images revealed a spherical morphology with a slightly smaller nanoparticle size (Figure 1B). As expected, this may be due to dehydrated nanoparticles compared with the hydrodynamic size observed in DLS.

**Figure 2:**
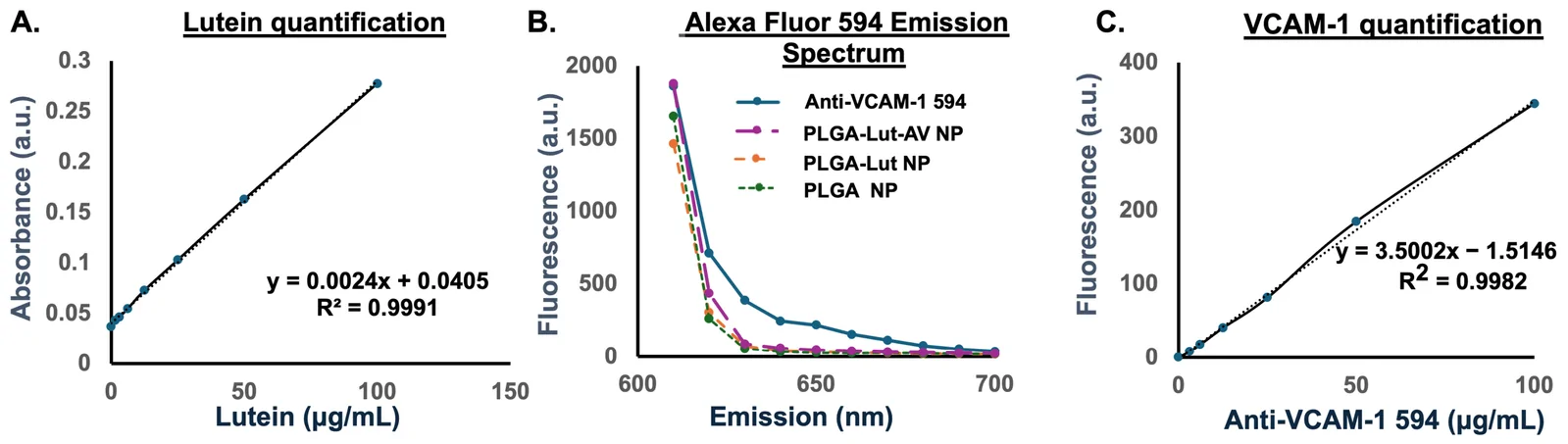
(**A**) An example calibration curve of the lutein standards was created at concentrations ranging from 0 to 100 μg/mL, measured at 490 nm absorption. From the resulting linear equation, the encapsulation was determined. The curve is generated each time nanoparticles are produced. (**B**) An example of the Alexa Fluor 594 VCAM-1 antibody emission spectrum. (**C**) An example calibration curve of the Alexa Fluor 594 VCAM-1 antibody standards was created at concentrations ranging from 0 to 100 μg/mL, measured at 590/617 nm excitation/emission. The curve is generated each time nanoparticles are produced. Using the resulting linear equation, the VCAM-1 amount in the PLGA-Lut-AV nanoparticles was determined.

Next, lutein encapsulation, VCAM-1 coating, and lutein release were characterized. First, a lutein calibration curve was generated at 490 nm absorbance, and the amount of lutein in the nanoparticles was quantified. The calibration curve was repeated each time a new formulation was produced. An example calibration curve is shown in (Figure 2A). As for VCAM-1, fluorescently labeled VCAM-1 was used to determine surface conjugation. Alexa Fluor 594 conjugated VCAM-1 was used, and the amount of VCAM-1 in the nanoparticles was determined by 590/617 nm excitation/emission. Similar to lutein detection, a new calibration curve was generated for each new formulation. An example Alexa Fluor-594-conjugated VCAM-1 emission spectrum and calibration curve are shown in Figure 2B,C. For lutein encapsulation, the efficiency was found to be around 10.67 ± 1.82%, while the VCAM-1 conjugation efficiency was found to be around 37.02 ± 3.62%. Further, the results reveal that almost 60% of the lutein was released after 24 h. The summary of the encapsulation, coating, and release results is shown in Table 1.

**Figure 3:**
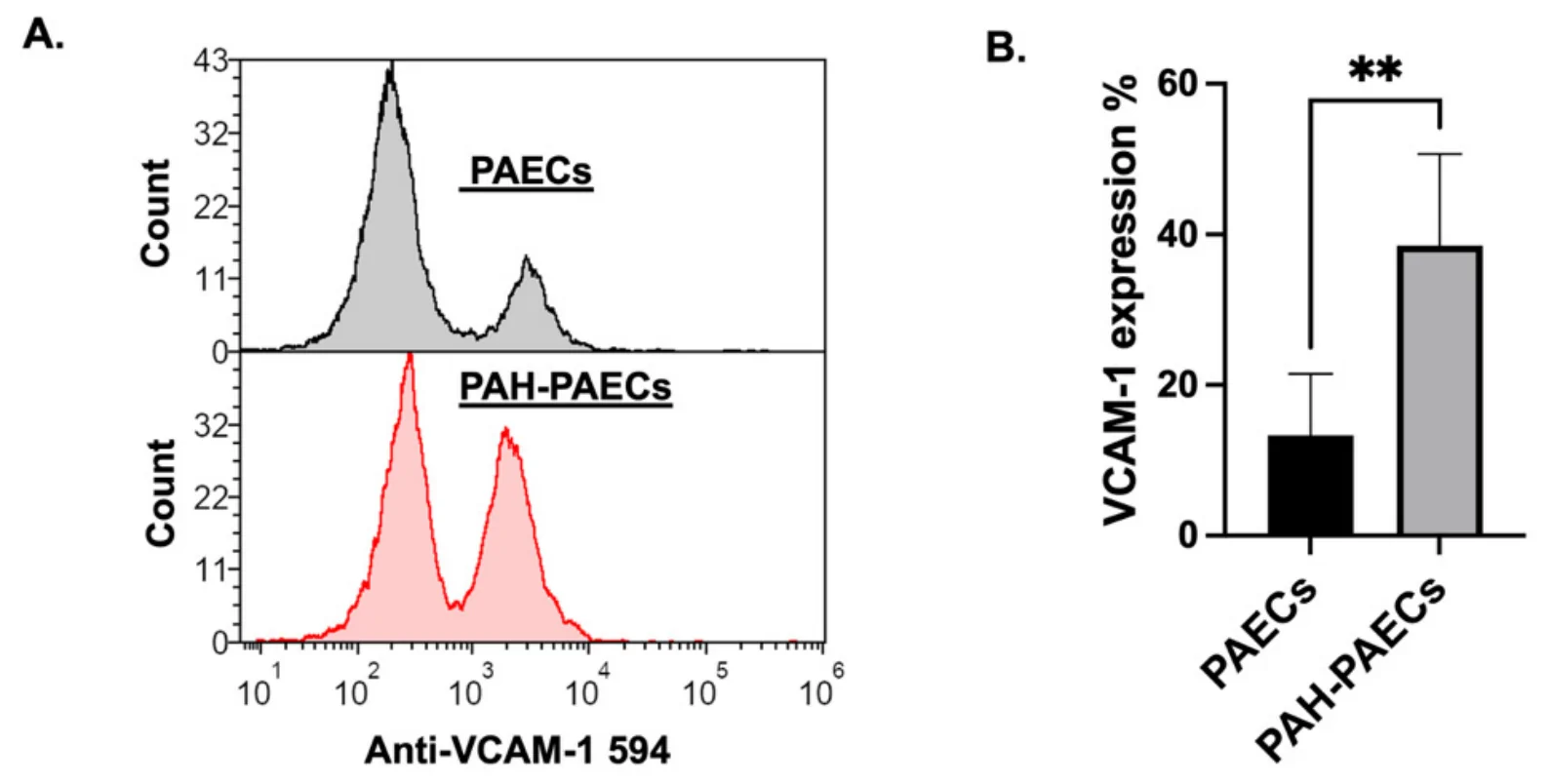
(**A**) VCAM-1 expression levels in healthy PAECs and PAH-PAECs analyzed by flow cytometry. The data depict VCAM-1 expression levels observed in healthy PAECs and PAH-PAECs. (**B**) Quantification of VCAM-1 expression. A threshold value of 700 was used for VCAM-1 expression. Cells with no antibody staining were used as a control. Data is represented as mean ± SEM (*n* = 3). Statistical significance: ** *p* < 0.01.

**Figure 4:**
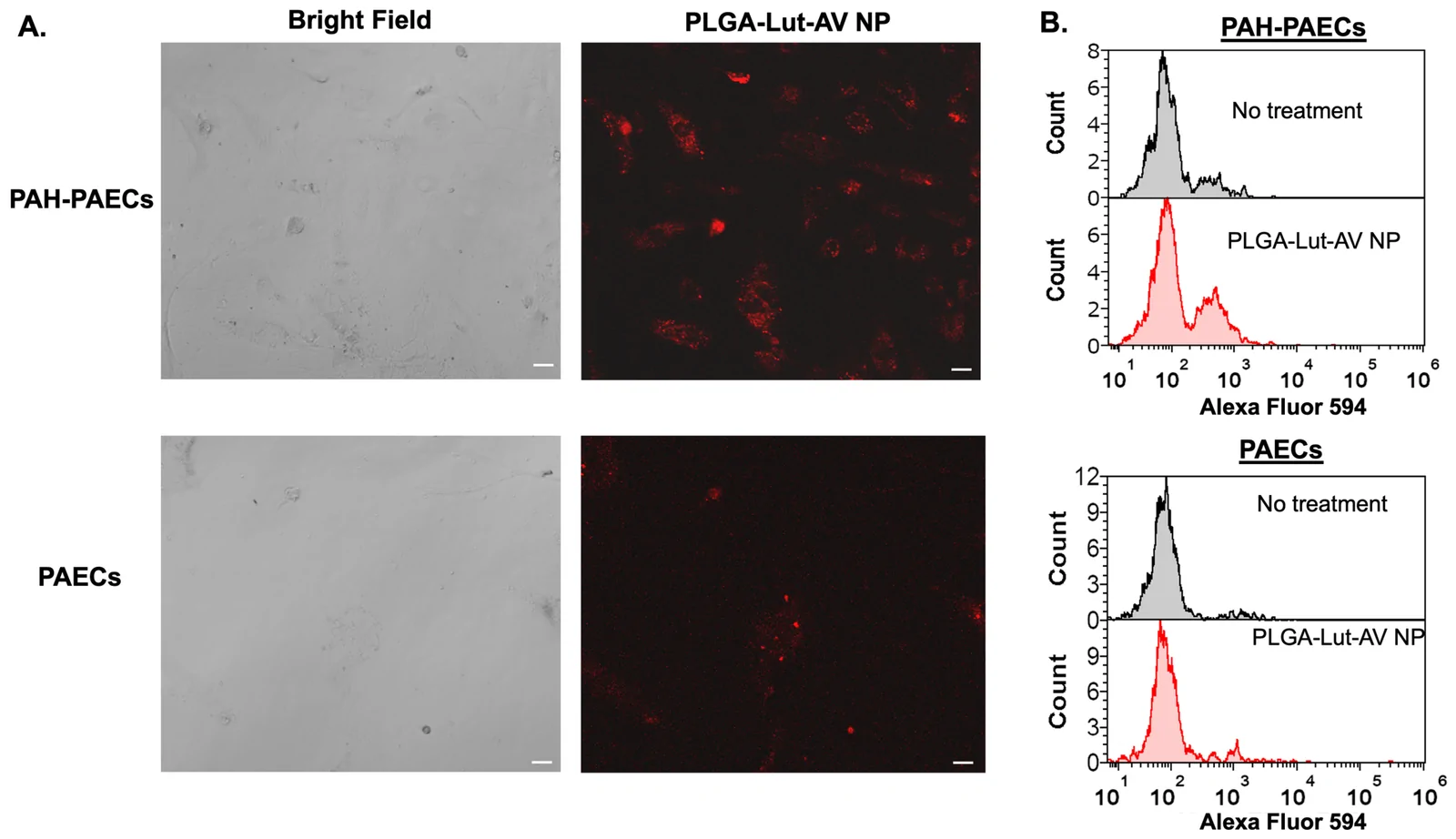
PLGA-Lut-AV nanoparticle uptake by healthy PAECs and PAH-PAECs. (**A**) Confocal images showing uptake of the PLGA-Lut-AV Nanoparticle. More particle uptake was observed in PAH-PAECs compared to healthy PAECs. Scale bar 50 μm. (**B**) Flow cytometry measurements of nanoparticle uptake by healthy PAECs and PAH-PAECs. A threshold value of 100 was used for quantification. The analysis shows significant uptake by PAH-PAECs compared to PAECs.

To test the effects of the nanoparticles on PAH-PAECs, we first tested nanoparticle EC targeting specificity. Both healthy and patient-derived PAECs were cultured in 24-well plates and, after 24 h, stained with Alexa Fluor 594 VCAM-1 antibody. The amount of VCAM-1 expression was assessed by flow cytometry (Figure 3). As shown in Figure 3B, VCAM-1 is highly expressed in patient-derived ECs compared with healthy ECs (Figure 3A). A threshold value of 700 was used for VCAM-1 expression, and analysis reveals 38.51 ± 5.43 percent of PAH-PAECs expressing VCAM-1 compared with 13.32 ± 4.07 percent in PAECs, indicating VCAM-1 to be a good targeting moiety for PAH-PAECs. We then tested the uptake of the nanoparticles by ECs using confocal imaging and flow cytometry (Figure 4). Image analysis by ImageJ software reveals a two-fold increase in fluorescence in patient ECs compared with healthy ECs (Figure 4A). Uptake analysis by flow cytometry also showed significant uptake in patient ECs compared to healthy ECs (Figure 4B). A threshold value of 100 was used for uptake quantification. Flow cytometry analysis reveals 29.85 ± 3.27 percent of patient ECs expressing VCAM-1, compared with 8.66 ± 3.25 percent in healthy ECs. The results indicate VCAM-1 to be a good targeting moiety for PAH-PAECs.

**Figure 5:**
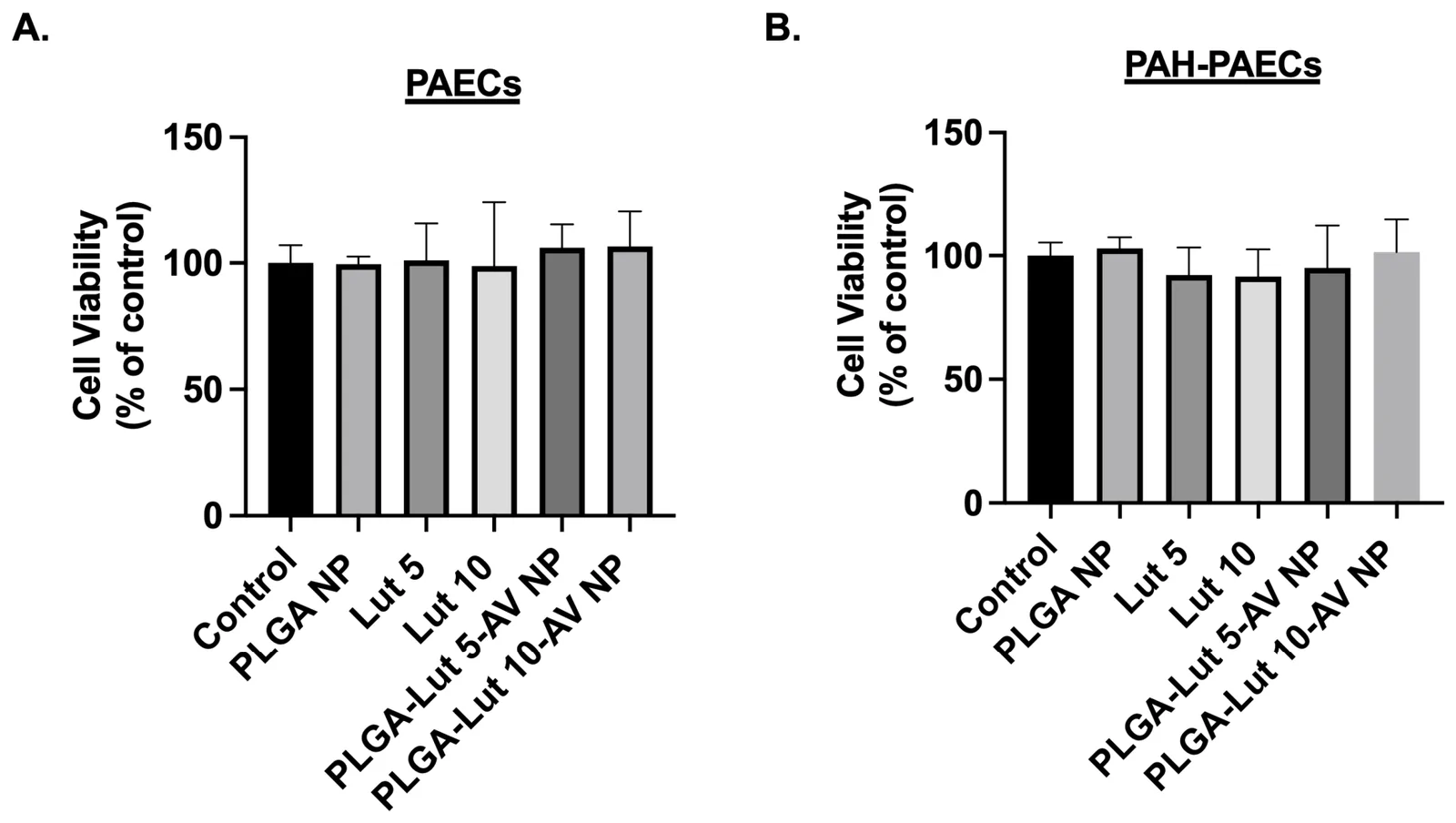
Effect of nanoparticles on the cell viability of (**A**) PAECs and (**B**) PAH-PAECs. Cell viability was measured by alamarBlue at 570/590 nm excitation/emission using a spectrophotometer. All treatments in healthy and patient cells exhibited cell viability above 95% with no significant differences compared to cells without any treatment (control), confirming that the nanoparticles are non-toxic. Data are shown as the mean ± SEM (*n* = 3).

**Figure 6:**
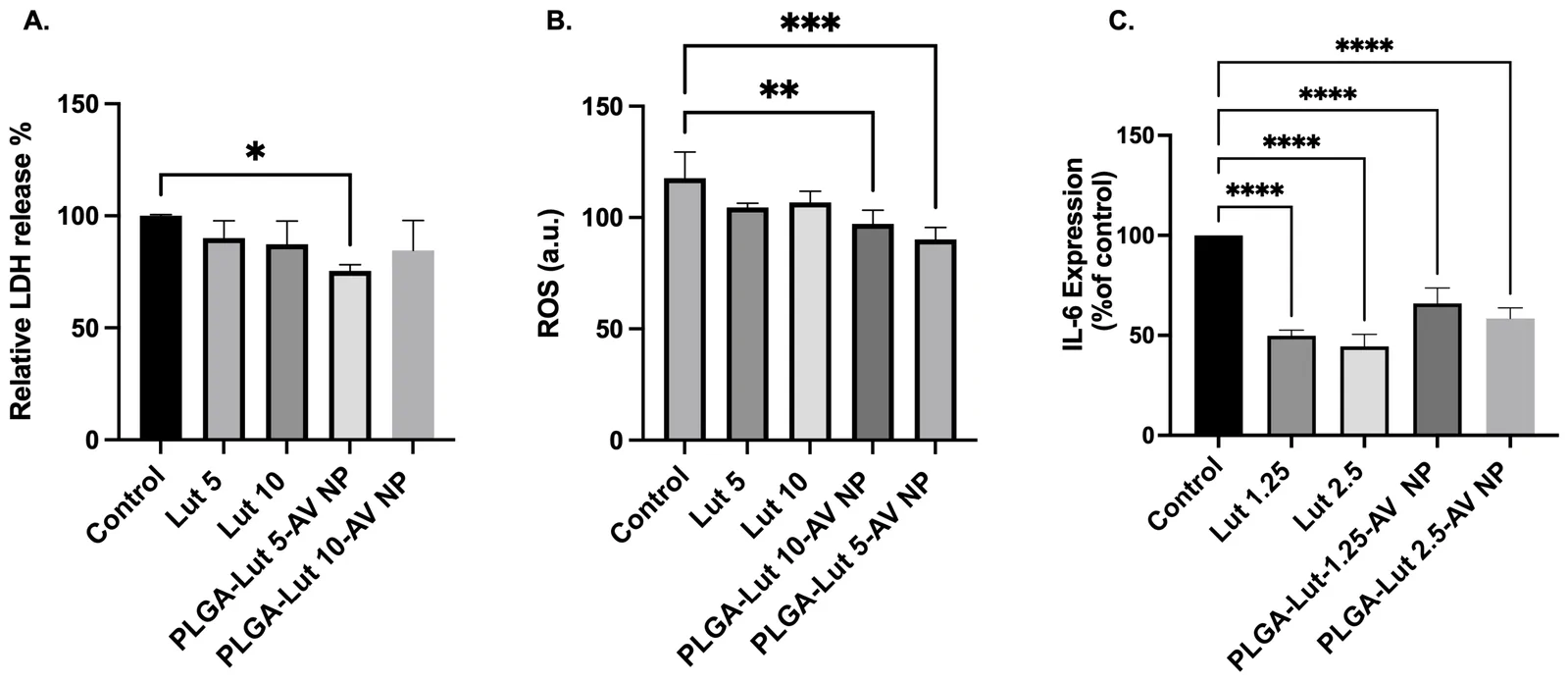
(**A**) LDH release in PAH-PAECs with and without free and/or PLGA-Lut-AV nanoparticle treatment was tested. Cells were seeded in a 96-well plate at a density of 10,000 cells/well. After 24 h, nanoparticles with lutein and lutein alone were treated at 5 µg/mL and 10 µg/mL lutein concentrations. Spontaneous LDH was assessed. Data are shown as the mean ± SEM (*n* = 3); statistical significance: * *p* < 0.05. (**B**) ROS production in PAH-PAECs with and without treatment. PLGA-Lut-AV Nanoparticle showed significant ROS inhibition among the treatment conditions. Data are displayed as the mean ± SEM (*n* = 3). Statistical significance: ** *p* < 0.01, *** *p* < 0.001. (**C**) Effect on inflammation was tested in PAH-PAECs. Cells were plated in 96-well plates at 10,000 cells/well and, after 24 h, treated with PLGA-Lut-AV nanoparticles or free lutein at 1.25 µg/mL and 2.5 µg/mL lutein concentrations. After 24 h, IL-6 levels were tested using a human IL-6 ELISA kit. Data are shown as the mean ± SEM (*n* = 3), statistical significance: **** *p* < 0.0001. Cells without any treatment were used as a control for the study.

To test whether the nanoparticles had any effect on cell viability, healthy PAECs and/or patient-derived ECs were treated with lutein, PLGA nanoparticles, and/or PLGA-Lut-AV nanoparticles at 5 and/or 10 μg/mL to achieve an equivalent lutein concentration. The results indicate no significant effect on cell viability in both healthy and patient PAECs (Figure 5A,B). We next investigated whether the nanoparticles modulate the cytotoxicity effects of PAH-PAECs by testing the LDH release levels with free lutein and/or PLGA-Lut-AV nanoparticles at 5 and/or 10 μg/mL of equivalent lutein concentration. PLGA-Lut-AV nanoparticles containing 5 μg/mL of lutein showed a significant reduction in LDH release compared to the PAH-PAECs without any treatment, indicating the nanoparticles’ ability to reduce cytotoxicity effects (Figure 6A). No significant effect on LDH release was observed at 10 μg/ml of lutein concentrations. Next, oxidative stress effects were assessed by ROS measurement. PAH-PAECs were treated with lutein and/or PLGA-Lut-AV nanoparticles, and the ROS measurement was carried out by Amplex Red assay at 560/590 nm excitation/emission. Results showed that both lutein and/or lutein nanoparticle treatment exhibited ROS inhibition (Figure 6B). Further, lutein PLGA-Lut-AV nanoparticles showed significant ROS inhibition compared to lutein alone. Finally, we used an IL-6 ELISA assay to assess whether the PLGA-Lut-AV nanoparticles exhibit anti-inflammatory properties. Both free lutein and lutein nanoparticles significantly reduced IL-6 levels compared to the PAH-PAECs without any treatment, indicating the anti-inflammatory properties of the formulation (Figure 6C).

## 3. Discussion

In this study, we developed PLGA-Lut-AV nanoparticles to target PAH-PAECs. Nanoparticles have the advantage of their targetability, bioavailability, and potential to encapsulate and solubilize hydrophobic drugs. PLGA, a widely used biomedical polymer, is hydrophobic and an excellent carrier for hydrophobic drug molecules like lutein. The current standard vasodilators are delivered systemically, which results in side effects. During PAH, several adhesion molecules are overexpressed, and these unique changes could be exploited to target the drug to the lung parenchyma. PAH-PAECs have been shown to overexpress the VCAM-1 molecule [14], and the nanoparticles formulated with VCAM-1 showed enhanced uptake compared to normal cells.

The PLGA-Lut-AV nanoparticles developed in this study exhibited a size of around 170 nm by DLS measurement, and TEM images showed spherical morphology typically observed for PLGA nanoparticles. Lutein release from the particles revealed about 60% release after 24 h. VCAM-1 overexpression was confirmed in PAH-PAECs compared to healthy PAECs. Nanoparticle targeting of PAH-PAECs and cellular uptake compared to healthy cells was confirmed by flow cytometry and confocal imaging. Further, PLGA-Lut-AV nanoparticles showed no significant effect on cell viability in both healthy and patient-derived ECs. Moreover, when PAH-PAECs were treated with the lutein nanoformulations, they exhibited significant antioxidant and anti-inflammatory properties by reducing ROS and IL-6 expression in PAH-PAECs compared to cells without any treatment.

Limitations include lutein stability during nanoparticle production and formulation. Lutein is extremely hydrophobic and sensitive to temperature and light. The current formulation was carried out over a period of 48 h, which could reduce the activity of lutein significantly. Future formulations should focus on a one-day formulation process consisting of 2–4 h of nanoprecipitation followed by a 2 h conjugation reaction, which could significantly reduce overall reaction time and improve lutein stability. Further, the lutein encapsulation efficiency could be further improved by tuning the formulation conditions. Another limitation is that although VCAM-1 antibody showed good targetability, antibodies are known to induce immune response. Due to the fragment crystallizable (F_C_) domain in the antibody structure, which is known to activate the complement system, the particles may get cleared by the immune system rapidly [31,32,33]. Hence, the formulation can be further modified with polyethylene glycol (PEG) [34] or cellular membranes [35] to increase circulation time and to have a stealth formulation. Further, shorter peptides or antibody fragments could be other alternatives for targeting moieties for clinical applications.

## 4. Materials and Methods

PLGA polymer, lutein (Lut), EmbryoMax 0.1% Gelatin solution, 1-Ethyl-3-(3-dimethylaminopropyl) carbodiimide hydrochloride (EDC)/N-hydroxysulfosuccinimide (NHS), poly vinyl alcohol (PVA), dimethylformamide (DMF), and phosphotungstic acid solution were obtained from Millipore Sigma (Milwaukee, WI, USA). Endothelial cell medium was purchased from ScienCell Research Laboratories (Carlsbad, CA, USA). Fetal bovine serum, antibiotic–antimycotic solution, and Tween 80 were purchased from Thermo Fisher Scientific (Waltham, MA, USA). The human IL-6 ELISA kit, LDH cytotoxicity assay, Amplex Red hydrogen peroxide/peroxidase assay, and alamarBlue were obtained from Invitrogen (Waltham, MA, USA). VCAM-1(B-K9) and VCAM-1-Alexa Fluor 594 (B-K9) antibodies were obtained from Novus Biologicals (Centennial, CO, USA).

### 4.1. Nanoparticle Production

The nanoprecipitation method was used to form the nanoparticles. First, PLGA polymer (10 mg) and lutein (1 mg) were dissolved in 1 mL dimethylformamide (DMF) and added dropwise to water into 9 mL water containing 0.15% (*w*/*w*) polyvinyl alcohol (PVA). Next, the particles were allowed to form overnight at room temperature, with gentle stirring, in the dark. Subsequently, the particles were purified by centrifugation at 10,000× *g*, 20 min, 4 °C. Next, the particles were surface functionalized with VCAM-1 antibody or VCAM-1-Alexa Fluor 594 antibody (AV) using 1-ethyl-3-(3-dimethylaminopropyl)carbodiimide/N-hydroxysulfosuccinimide (EDC/NHS) chemistry in phosphate buffer (PBS), pH 7.4, with slight modification from previously reported methods [36,37]. The reaction was carried out overnight at 4 °C, and next the VCAM-1-coated nanoparticles (PLGA-Lut-AV) were purified by centrifugation at 10,000× *g*, 20 min, 4 °C and then physicochemical characterization was performed.

### 4.2. Nanoparticle Characterization

Size and morphology: Nanoparticle size and morphology were assessed by dynamic light scattering (DLS) and transmission electron microscopy (TEM). For the DLS measurement, nanoparticles were diluted in water, and size measurements were performed using a Malvern Zetasizer instrument (Westborough, MA, USA) in the lab. For TEM imaging, 5 µL of nanoparticles were spotted on a 400-mesh copper grid (Electron Microscopy Sciences, Morgantown, PA, USA) and stained with 2% phosphotungstic acid (pH 7.4). Imaging was performed using JEOL TEM at the University of Michigan Ann Arbor Medical School’s Electron Microscopy Facility.

Encapsulation, surface coating, and release: Lutein amount in the nanoparticles was determined by absorption at 490 nm. A calibration curve was generated using lutein standards at concentrations ranging from 0 to 100 μg/mL. The curve was generated each time the nanoparticles were produced. Nanoparticles were degraded with 6 M NaOH, and the amount of lutein in the nanoparticles was measured by absorbance at 490 nm and subsequently calculated based on the curve. To determine the amount of VCAM-1, Alexa Fluor-594-labeled VCAM-1 was used for the conjugation reaction. Calibration curves were generated, and the amount of VCAM-1 on the nanoparticles was determined by Alexa Fluor-594 fluorescence at 590/617 nm excitation/emission. To determine the amount of lutein released over time, PLGA-Lut-AV nanoparticles (100 μL) were dialyzed in a 3.5 K Slide-A-Lyzer dialysis device 3.5 K MWCO (Thermo Fisher Scientific; Waltham, MA, USA) in 15 mL of PBS containing 0.1% Tween 80. Release was carried out at 37 °C under stirring for 24 h. Lutein release was measured at 2 h and 24 h. Release amount was calculated based on lutein absorbance at 490 nm, as mentioned above.

### 4.3. Nanoparticle Targeting and Uptake

De-identified human PAH patient-derived ECs (PAH-PAECs) and healthy control ECs were obtained from the Pulmonary Hypertension Breakthrough Initiative (PHBI) Network. The study using these de-identified human cells has been determined and approved as Non-Human Subjects Research by the Medical College of Wisconsin Institutional Review Board (IRB PRO00029154). We only obtained de-identified cells along with clinical/phenotype data that does not include identifiers, and therefore the privacy of patients and confidentiality of data have been strictly protected. Cells were cultured in complete EC medium (Scientific Research Laboratories, Carlsbad, CA, USA), with an additional 5% FBS, 1% L-glutamine, and 1% antibiotic/antimycotic. To assess VCAM-1 expression in the cells, healthy PAECs and PAH-PAECs were cultured in 24-well plates at 75,000 cells/well in 500 μL of media for 24 h. Next, cells were trypsinized and fixed with 4% paraformaldehyde (PFA). Cells were then permeabilized with 0.1% Triton-x, stained with Alexa Fluor 594 VCAM-1 antibody, and analyzed using an Attune Flow Cytometer in the lab. Next, the uptake of the nanoparticles by the cells was studied. Cells were cultured in 24-well plates at 50,000 cells/well in 500 μL of media for 24 h. Next, the cells were treated with PLGA-Lut-AV nanoparticles for 30 min, and the uptake was measured. Confocal imaging was performed using an Olympus laser scanning confocal microscope (FV1200) (Needham, MA, USA) at UM-Dearborn. Attune flow cytometer data was analyzed by FCS Express 7 Research (Version 7.28.0035) software, and confocal fluorescence images were analyzed by ImageJ 1.54q software.

### 4.4. Cellular Efficacy Studies

Cell viability: Cell viability was tested using alamarBlue. Healthy and PAH-PAECs were cultured in 96-well plates at 10,000 cells/well in 100 μL of media for 24 h in EC medium (ScienCell Research Laboratories). Subsequently, cells were treated with lutein and/or PLGA-Lut-AV nanoparticles at 5 and 10 μg/mL (equivalent lutein concentrations) for 24 h. Next, alamarBlue was added to the cells, and after 2 h, cell viability was measured at 570/590 nm excitation/emission using an M3 spectrophotometer.

LDH Release Assay: Cellular toxicity was tested using the LDH Release Assay. PAH-PAECs were plated under similar conditions used for the viability assay and treated with lutein and PLGA-Lut-AV nanoparticles at 5 and 10 μg/mL equivalent concentrations of lutein for 24 h. LDH assay was performed according to the manufacturer’s protocol. LDH release was compared with the control under treated and untreated conditions.

Oxidative stress (Amplex Red ROS assay): The levels of reactive oxygen species (ROS) were measured to determine the antioxidative properties of the nanoparticles. PAH-PAECs were cultured in 24 wells at a density of 50,000 cells/well. Cells were then treated with PLGA-Lut-AV nanoparticles at 5 and 10 μg/mL equivalent concentrations of lutein after 24 h, and an Amplex Red assay was performed. ROS effects were measured at 560/590 excitation/emission and assessed by a spectrophotometer.

IL-6 ELISA assay: The assay was performed to assess the anti-inflammatory properties of the nanoparticles. PAH-PAECs were cultured in 96 wells at a density of 10,000 cells/well in 100 μL of media for 24 h. Lutein and nanoparticles were added to cells to achieve a final lutein concentration of 2.5 and 5 μg/mL. After 24 h of treatment, 100 μL of media from each condition were taken for analysis with a human IL-6 ELISA kit. The test was performed according to the manufacturer’s protocol. Absorbance was measured at 450 nm. An IL-6 standard curve was created, and IL-6 concentrations for the experimental conditions were calculated accordingly.

All experiments and assays performed in this study were carried out at least three times independently.

Statistical analysis: At least three independent experiments were performed for each assay/test in this study. Data is presented as the mean standard error (S.E.M.). Prism software (version 9.20) and Microsoft Excel (version 16.69) were used for analysis. Statistical significance was assessed using Analysis of variance (ANOVA) followed by unpaired Student’s *t*-test or Tukey’s HSD post hoc test. Statistical significance represented as * *p* < 0.05, ** *p* < 0.01, *** *p* < 0.001, **** *p* < 0.0001.

## 5. Conclusions

In summary, the nanoparticle-based targeted drug delivery depot presented in this proof-of-principle study strengthens the role of nanocarriers as effective drug-delivery vehicles. By targeting PAH vasculature, the formulation could mitigate the side effects of systemic drug delivery while improving therapeutic efficacy. The technology could be applied to traditional vasodilators in combination with antioxidant and/or anti-inflammatory drugs to render a dual-targeting effect. However, to realize the translational potential of this technology, comprehensive long-term biocompatibility and biodistribution studies must be conducted in translationally relevant PAH models. Further, development of tunable nanoparticles and patient-specific nanoparticle delivery systems would have further impact on the treatment. Additionally, the targeting moieties could be modified with antibody fragments or peptides to minimize undesirable side effects and immune responses. Overall, nanotherapeutics offer a promising platform for treating PAH with minimal side effects and improved therapeutic efficacy.

## Figures and Tables

**Table 1:** PLGA-Lut-AV nanoparticle encapsulation, conjugation, and release data. Data are shown as the mean ± SEM (*n* = 3).

Nanoparticle	Lut Encapsulation Efficiency (%)	AV Conjugation Efficiency (%)	Drug Release (%)	
			2 h	24 h
PLGA-Lut-AV NP	10.67 ± 1.82	37.02 ± 3.62	25.88 ± 8.69	57.77 ± 6.67

## Data Availability

The data relevant to the results of the article will be made available by the authors without any restrictions.

## References

[1] Farber H.W., Loscalzo J. (2004). Pulmonary arterial hypertension. N. Engl. J. Med..

[2] Tuder R.M., Marecki J.C., Richter A., Fijalkowska I., Flores S. (2007). Pathology of pulmonary hypertension. Clin. Chest Med..

[3] Delgado-Rizo V., Martinez-Guzman M.A., Iniguez-Gutierrez L., Garcia-Orozco A., Alvarado-Navarro A., Fafutis-Morris M. (2017). Neutrophil Extracellular Traps and Its Implications in Inflammation: An Overview. Front. Immunol..

[4] Mutua V., Gershwin L.J. (2021). A Review of Neutrophil Extracellular Traps (NETs) in Disease: Potential Anti-NETs Therapeutics. Clin. Rev. Allergy Immunol..

[5] Aldabbous L., Abdul-Salam V., McKinnon T., Duluc L., Pepke-Zaba J., Southwood M., Ainscough A.J., Hadinnapola C., Wilkins M.R., Toshner M. (2016). Neutrophil Extracellular Traps Promote Angiogenesis: Evidence From Vascular Pathology in Pulmonary Hypertension. Arterioscler. Thromb. Vasc. Biol..

[6] Repine J.E., Bast A., Lankhorst I. (1997). Oxidative stress in chronic obstructive pulmonary disease. Oxidative Stress Study Group. Am. J. Respir. Crit. Care Med..

[7] Bezerra F.S., Lanzetti M., Nesi R.T., Nagato A.C., Silva C.P.E., Kennedy-Feitosa E., Melo A.C., Cattani-Cavalieri I., Porto L.C., Valenca S.S. (2023). Oxidative Stress and Inflammation in Acute and Chronic Lung Injuries. Antioxidants.

[8] Rabinovitch M. (2012). Molecular pathogenesis of pulmonary arterial hypertension. J. Clin. Investig..

[9] Guignabert C., Tu L., Girerd B., Ricard N., Huertas A., Montani D., Humbert M. (2015). New molecular targets of pulmonary vascular remodeling in pulmonary arterial hypertension: Importance of endothelial communication. Chest.

[10] Zijlstra W.M.H., Douwes J.M., Rosenzweig E.B., Schokker S., Krishnan U., Roofthooft M.T.R., Miller-Reed K., Hillege H.L., Ivy D.D., Berger R.M.F. (2014). Survival differences in pediatric pulmonary arterial hypertension: Clues to a better understanding of outcome and optimal treatment strategies. J. Am. Coll. Cardiol..

[11] Shah A.J., Beckmann T., Vorla M., Kalra D.K. (2023). New Drugs and Therapies in Pulmonary Arterial Hypertension. Int. J. Mol. Sci..

[12] Correale M., Mercurio V., Bevere E.M.L., Pezzuto B., Tricarico L., Attanasio U., Raucci A., Ferrara A.L., Loffredo S., Puteo C. (2025). Pathophysiology of Pulmonary Arterial Hypertension: Focus on Vascular Endothelium as a Potential Therapeutic Target. Int. J. Mol. Sci..

[13] Tobal R., Potjewijd J., van Empel V.P.M., Ysermans R., Schurgers L.J., Reutelingsperger C.P., Damoiseaux J., van Paassen P. (2021). Vascular Remodeling in Pulmonary Arterial Hypertension: The Potential Involvement of Innate and Adaptive Immunity. Front. Med..

[14] Le Hiress M., Tu L., Ricard N., Phan C., Thuillet R., Fadel E., Dorfmuller P., Montani D., de Man F., Humbert M. (2015). Proinflammatory Signature of the Dysfunctional Endothelium in Pulmonary Hypertension. Role of the Macrophage Migration Inhibitory Factor/CD74 Complex. Am. J. Respir. Crit. Care Med..

[15] Ezike T.C., Okpala U.S., Onoja U.L., Nwike C.P., Ezeako E.C., Okpara O.J., Okoroafor C.C., Eze S.C., Kalu O.L., Odoh E.C. (2023). Advances in drug delivery systems, challenges and future directions. Heliyon.

[16] Mitchell M.J., Billingsley M.M., Haley R.M., Wechsler M.E., Peppas N.A., Langer R. (2021). Engineering precision nanoparticles for drug delivery. Nat. Rev. Drug Discov..

[17] Shi J., Votruba A.R., Farokhzad O.C., Langer R. (2010). Nanotechnology in drug delivery and tissue engineering: From discovery to applications. Nano Lett..

[18] Brenner J.S., Greineder C., Shuvaev V., Muzykantov V. (2015). Endothelial nanomedicine for the treatment of pulmonary disease. Expert Opin. Drug Deliv..

[19] Khalid-Salako F., Salimi Khaligh S., Fathi F., Demirci O.C., Oncer N., Kurt H., Yuce M. (2025). The Nanocarrier Landscape horizontal line Evaluating Key Drug Delivery Vehicles and Their Capabilities: A Translational Perspective. ACS Appl. Mater. Interfaces.

[20] Danhier F., Ansorena E., Silva J.M., Coco R., Le Breton A., Preat V. (2012). PLGA-based nanoparticles: An overview of biomedical applications. J. Control. Release.

[21] Omidian H., Wilson R.L. (2025). PLGA-Based Strategies for Intranasal and Pulmonary Applications. Pharmaceutics.

[22] Li Y., Tao T., Xiong Y., Guo W., Liang Y. (2025). Multifunctional PLGA nanosystems: Enabling integrated diagnostic and therapeutic strategies. Front. Pharmacol..

[23] Ahn Y.J., Kim H. (2021). Lutein as a Modulator of Oxidative Stress-Mediated Inflammatory Diseases. Antioxidants.

[24] Chung R.W.S., Leanderson P., Lundberg A.K., Jonasson L. (2017). Lutein exerts anti-inflammatory effects in patients with coronary artery disease. Atherosclerosis.

[25] Izumi-Nagai K., Nagai N., Ohgami K., Satofuka S., Ozawa Y., Tsubota K., Umezawa K., Ohno S., Oike Y., Ishida S. (2007). Macular pigment lutein is antiinflammatory in preventing choroidal neovascularization. Arterioscler. Thromb. Vasc. Biol..

[26] Wynants M., Vengethasamy L., Ronisz A., Meyns B., Delcroix M., Quarck R. (2013). NF-kappaB pathway is involved in CRP-induced effects on pulmonary arterial endothelial cells in chronic thromboembolic pulmonary hypertension. Am. J. Physiol. Lung Cell. Mol. Physiol..

[27] Zhao C., Shen X., Guo M. (2018). Stability of lutein encapsulated whey protein nano-emulsion during storage. PLoS ONE.

[28] Bhat I., Jose N.M., Mamatha B.S. (2023). Oxidative stability of lutein on exposure to varied extrinsic factors. J. Food Sci. Technol..

[29] Algan A.H., Gungor-Ak A., Karatas A. (2022). Nanoscale Delivery Systems of Lutein: An Updated Review from a Pharmaceutical Perspective. Pharmaceutics.

[30] Nguyen D., Sarkar S., Thrimawithana T., Piva T., Conn C.E., Luu C., Huynh T. (2025). Optimizing Lutein Formulations for Targeted Ocular Drug Delivery: In Vitro and In Vivo Insights. ACS Appl. Mater. Interfaces.

[31] Glassman P.M., Myerson J.W., Ferguson L.T., Kiseleva R.Y., Shuvaev V.V., Brenner J.S., Muzykantov V.R. (2020). Targeting drug delivery in the vascular system: Focus on endothelium. Adv. Drug Deliv. Rev..

[32] Ahmad Z.A., Yeap S.K., Ali A.M., Ho W.Y., Alitheen N.B., Hamid M. (2012). scFv antibody: Principles and clinical application. J. Immunol. Res..

[33] Todaro B., Ottalagana E., Luin S., Santi M. (2023). Targeting Peptides: The New Generation of Targeted Drug Delivery Systems. Pharmaceutics.

[34] Cui J., Bjornmalm M., Ju Y., Caruso F. (2018). Nanoengineering of Poly(ethylene glycol) Particles for Stealth and Targeting. Langmuir.

[35] Jan N., Rejili M. (2026). Nature-Inspired Alternatives to PEG for Next-Generation Long-Circulating Nanocarriers. Int. J. Nanomed..

[36] Rafat M., Rotenstein L.S., Hu J.L., Auguste D.T. (2012). Engineered endothelial cell adhesion via VCAM1 and E-selectin antibody-presenting alginate hydrogels. Acta Biomater..

[37] Almeda D., Wang B., Auguste D.T. (2015). Minimizing antibody surface density on liposomes while sustaining cytokine-activated EC targeting. Biomaterials.

